# Deep Learning-Based Drug Compounds Discovery for Gynecomastia

**DOI:** 10.3390/biomedicines13020262

**Published:** 2025-01-21

**Authors:** Yeheng Lu, Byeong Seop Kim, Junhao Zeng, Zhiwei Chen, Mengyu Zhu, Yuxi Tang, Yuyan Pan

**Affiliations:** 1 Department of Plastic and Reconstructive Surgery, Zhongshan Hospital, Fudan University, Shanghai 200032, China; luyeheng97@126.com (Y.L.); noahzeng1999@163.com (J.Z.); 2 Department of Plastic and Reconstructive Surgery, Shanghai Ninth People’s Hospital, School of Medicine, Shanghai Jiaotong University, Shanghai 200011, China; supki3@icloud.com; 3 Big Data and Artificial Intelligence Center, Zhongshan Hospital, Fudan University, Shanghai 200032, China; chen.zhiwei@zs-hospital.sh.cn; 4 School of Medicine, Shanghai Jiao Tong University, Shanghai 200025, China; molliez@163.com

**Keywords:** gynecomastia, drug–target interactions (DTIs), DeepPurpose, deep learning (DL), drug therapy

## Abstract

**Background:** Gynecomastia, caused by an estrogen–testosterone imbalance, affects males across various age groups. With unclear mechanisms and no approved drugs, the condition underscores the need for efficient, innovative treatment strategies. **Methods:** This study utilized deep learning-based computational methods to discover potential drug compounds for gynecomastia. To identify genes and pathways associated with gynecomastia, initial analyses included text mining, biological process exploration, pathway enrichment and protein–protein interaction (PPI) network construction. Subsequently, drug–target interactions (DTIs) were examined to identify potential therapeutic compounds. The DeepPurpose toolkit was employed to predict interactions between these candidate drugs and gene targets, prioritizing compounds based on their predicted binding affinities. **Results:** Text mining identified 177 genes associated with gynecomastia. Gene Ontology (GO) biological process and Kyoto Encyclopedia of Genes and Genomes (KEGG) pathway analyses identified critical genes and pathways, with notable involvement in signal transduction, cell proliferation and steroid hormone biosynthesis. PPI network analysis highlighted 10 crucial genes, such as IGF1, TGFB1 and AR. DTI analysis and DeepPurpose predictions identified 12 potential drugs, including conteltinib, yifenidone and vosilasarm, with high predicted binding affinities to the target genes. **Conclusions:** The study successfully identified potential drug compounds for gynecomastia using a deep learning-based approach. The findings highlight the effectiveness of combining text mining and artificial intelligence in drug discovery. This innovative method provides a new avenue for developing specific treatments for gynecomastia and underscores the need for further experimental validation and optimization of prediction models to support novel drug development.

## 1. Introduction

Gynecomastia typically arises from an imbalance between estrogen and testosterone levels in men, leading to the abnormal growth of glandular breast tissue. Clinically, it should be distinguished from pseudogynecomastia and breast carcinoma before leaping into a firmly established diagnosis [1]. However, such enlargement of the glandular tissue has troubled men for a long time, posing potential health threats. Gynecomastia is most prevalent during infancy, adolescence and later adulthood [2]. The prevalence rates are between 60 and 90% in newborns, 50 and 60% in adolescents and 70% in men aged 50 to 69 years [3]. Pathologic gynecomastia can occur at any age as a consequence of various medical conditions, medication or substance use, including the long-term use of antipsychotics and antiretrovirals. 

Although most patients with gynecomastia do not require treatment due to minimal symptomatic concerns, various therapeutic options, including medication, surgical subcutaneous mastectomy, ultrasound-assisted liposuction and suction-assisted lipectomy, have been explored in clinical practice [4]. Because of the limited randomized, double-blinded, placebo-controlled trials that have been carried out and the spontaneous resolution of gynecomastia, most studies of drugs (such as testosterone and dihydrotestosterone) have been uncontrolled and difficult to interpret. Nevertheless, both doctors and patients are faced with such a dilemma that there is no specific approved drug for the treatment of gynecomastia. For current therapies, adverse events still cannot be avoided, with patients receiving unsatisfactory effectiveness. Meanwhile, the underlying mechanism of gynecomastia remains unclear, making efforts to combat gynecomastia a persistent challenge. 

Drug discovery remains a resource-intensive and time-consuming process, despite advances in recent decades, with expenses for new molecular entities estimated at USD 1.8 billion [5,6,7]. However, the traditional drug discovery system is limited to the one molecule–one target–one disease paradigm, neglecting the multifactorial complexity of disease. In recent years, accelerating drug discovery has become increasingly urgent, necessitating precise identification of the complex interactions between drugs and diverse protein targets, underscoring the critical importance of drug–target interactions (DTIs) [8,9].

Deep learning (DL) technology through computational prediction methods appears more appropriate and powerful in the field of DTI prediction compared with time-consuming and expensive experimental verification, as it can analyze complex drug–target associations effectively [10,11]. DeepPurpose is a deep learning model designed to analyze drug–target affinity. It employs a coding–decoding architecture that utilizes various vector embedding techniques to transform sequence-based sparse features into dense vector representations. By leveraging multiple deep neural networks, the model automatically extracts features for both drugs and targets. With support for over 50 deep learning models, seven protein encoders and eight compound encoders, DeepPurpose facilitates efficient prototyping through its programming framework, enabling accurate prediction of drug-target affinity [12].

This study aimed to identify novel therapeutic options for gynecomastia using computational approaches. Initially, text mining, biological process and pathway analysis, and protein–protein interaction (PPI) network analysis were conducted to identify target genes and pathways closely associated with gynecomastia. Subsequently, DTI analysis was employed to pinpoint potential candidate drugs. Finally, DeepPurpose, an advanced Python-based toolkit, was utilized to predict interactions between candidate drugs and gene targets, prioritizing drugs based on their predicted binding affinities from a ranked list.

## 2. Materials and Methods

### 2.1. Text Mining

Through pubmed2ensembl (http://pubmed2ensembl.ls.manchester.ac.uk (accessed on 31 July 2022)), prime information was collected from numerous biological studies [13]. Gynecomastia was used as the search term. “*Homo sapiens*” was selected as the species dataset, before “Ensembl Gene ID” and “Associated Gene Name” were chosen under the GENE category. “Search for PubMed IDs” and “filter on Entrez: PMID” drop-down menus were utilized for each query search. The output gene list was saved for further analysis. The study adhered to the principles outlined in the Declaration of Helsinki.

### 2.2. Biological Process and Pathway Analysis

Enrichment analysis of genes closely associated with gynocomastia was conducted using GeneCodis (http://genecodis.genyo.es/ (accessed on 31 August 2022)) [14]. Initially, genes identified through text mining were analyzed using Gene Ontology (GO) biological process enrichment. Subsequently, the most significantly enriched genes in biological processes were selected for Kyoto Encyclopedia of Genes and Genomes (KEGG) pathway analysis. Key KEGG pathways with the highest enrichment were identified, and their associated genes were retained for further investigation.

### 2.3. Protein–Protein Interaction Network

A PPI network was constructed to visually represent the genes identified in the previous step, utilizing the STRING database (Search Tool for the Retrieval of Interacting Genes/Proteins, http://string-db.org (accessed on 31 August 2022)) [15]. The identified genes were entered under the “Multiple proteins” menu, and “*Homo sapiens*” was selected as the species dataset. To ensure high-quality interactions, a confidence score threshold of 0.700 was applied, resulting in the generation of a PPI network for the target genes. The centrality parameters (“degree” and “betweenness”) of the PPI network were then determined by the CentiScape plugin in Cytoscape (The Cytoscape Consortium, San Diego, CA, USA) [16]. Regarding the two key parameters, degree represents the total number of edges connected to a node, while betweenness measures the number of shortest paths that pass through the node.

### 2.4. Drug-Gene Interactions

Through Pharmaprojects (https://pharmaintelligence.informa.com/ (Citeline, New York, NY, USA) (accessed on 31 October 2022)), drugs targeting genes strongly associated with gynecomastia were identified and collected [17]. Each gene query can retrieve a drug list with detailed information including the global status, disease, mechanism of action, delivery route, target and chemical structure (SMILES format). Drugs with the global status of “launched”, “phase I/II/III clinical trial”, “pre-registration”, or “registered” were excluded. Meanwhile, those with the delivery route of “oral” or “oral, swallowed” were also eliminated. Using the aforementioned screening criteria, candidate drugs with strong targeting ability, rapid onset of action and minimal side effects were identified. Drugs obtained through such DTI analysis may serve as potential therapeutic options for gynecomastia.

### 2.5. DeepPurpose

First, the target proteins were translated into amino acid sequences along with SMILES fingerprints of the potential drugs. The binding affinity between each drug molecule and its corresponding protein target was evaluated using pre-trained models from DeepPurpose. To ensure comprehensive analysis, predictions were generated separately across 15 distinct models within DeepPurpose. Thresholds were applied to identify potential drug–target interactions with high confidence. These interactions were further validated against a curated validation set, after which binding affinity scores were combined using the aggregation method provided by DeepPurpose. Subsequently, a comparative analysis was then conducted to assess discrepancies between the predictions from individual models and those derived from the aggregated approach.

### 2.6. Statistical Analysis

Statistical analysis was performed using machine learning algorithms integrated within the DeepPurpose framework.

## 3. Results

### 3.1. Results of Text Mining, Biological Process and Pathway Analysis

**Figure 1:**
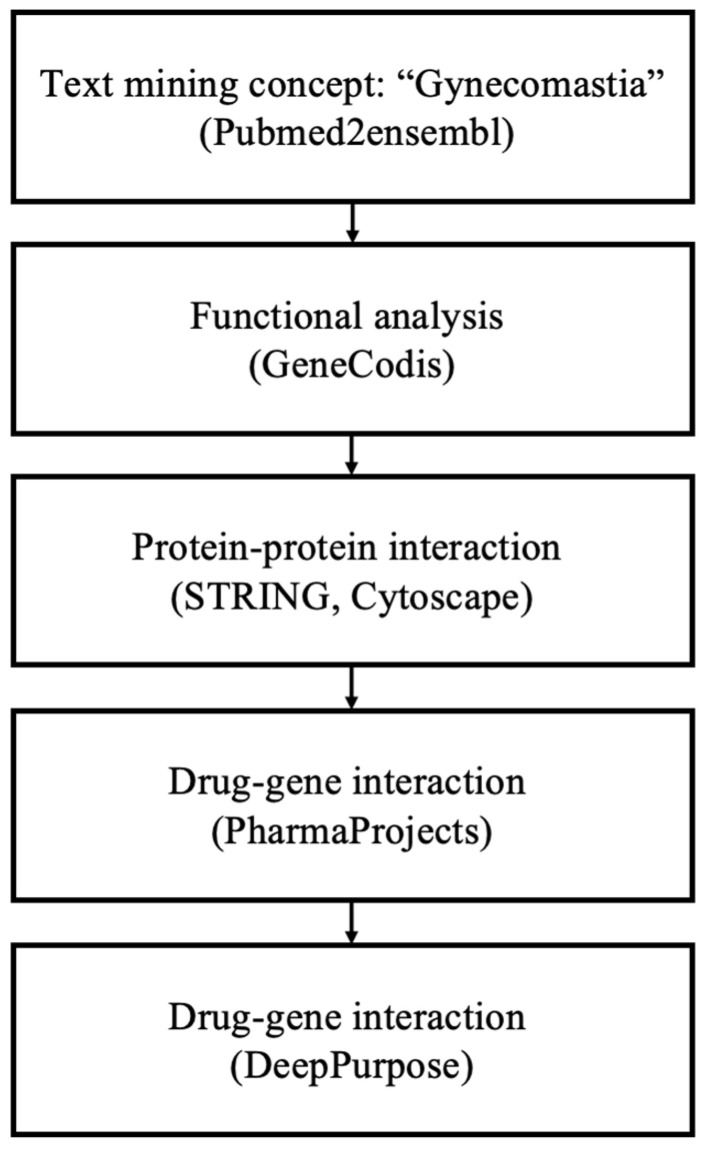
Overview of the data mining workflow.

Following the methodology outlined in Figure 1 for data mining, a total of 177 genes associated with ’gynecomastia’ were identified. Employing the GeneCodis tool, a GO biological process analysis was conducted using the aforementioned gene pool. This analysis validated the connection between the top enriched terms and the occurrence of gynecomastia. To balance the comprehensiveness and specificity of the GO biological process analysis, a significance threshold of *p* = 1.00 × 10^−8^ was chosen. Subsequently, 120 annotation sets, comprising 118 genes, were extracted from GeneCodis (Table 1). The top three significantly enriched biological processes were identified as follows: (1) ’signal transduction’ (*p* = 3.67 × 10^−23^), (2) ’positive regulation of cell population proliferation’ (*p* = 1.98 × 10^−21^) and (3) ’cell–cell signaling’ (*p* = 4.01 × 10^−21^), encompassing 40, 26 and 20 genes, respectively. Additional significantly enriched biological process annotations encompassed ’steroid biosynthetic process’, ’lipid metabolic process, steroid biosynthetic process’ and ’negative regulation of apoptotic process’.

Subsequently, a KEGG pathway analysis was executed, utilizing a *p*-value cutoff of *p* = 1.00 × 10^−12^, yielding 25 pathways and involving 72 genes (Table 2). The three most significantly enriched pathways were identified as (1) the ’PI3K-Akt signaling pathway’ (*p* = 2.49 × 10^−31^), (2) ’steroid hormone biosynthesis’ (*p* = 5.16 × 10^−27^) and (3) ’neuroactive ligand–receptor interaction’ (*p* = 4.05 × 10^−25^). Other enriched pathways included ’metabolic pathways, steroid hormone biosynthesis’, ’pathways in cancer’ and the ’MAPK signaling pathway’.

### 3.2. Results of Protein–Protein Interaction

**Figure 2:**
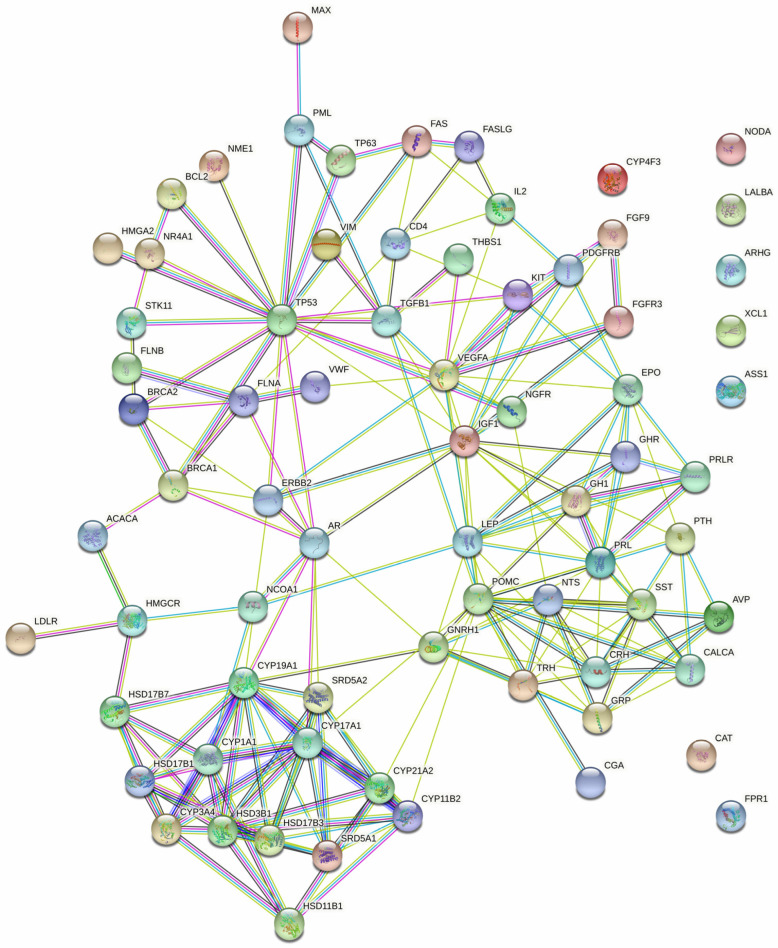
The protein–protein interaction network of the 54 targeted genes, generated using STRING with a confidence score = 0.700.

**Figure 3:**
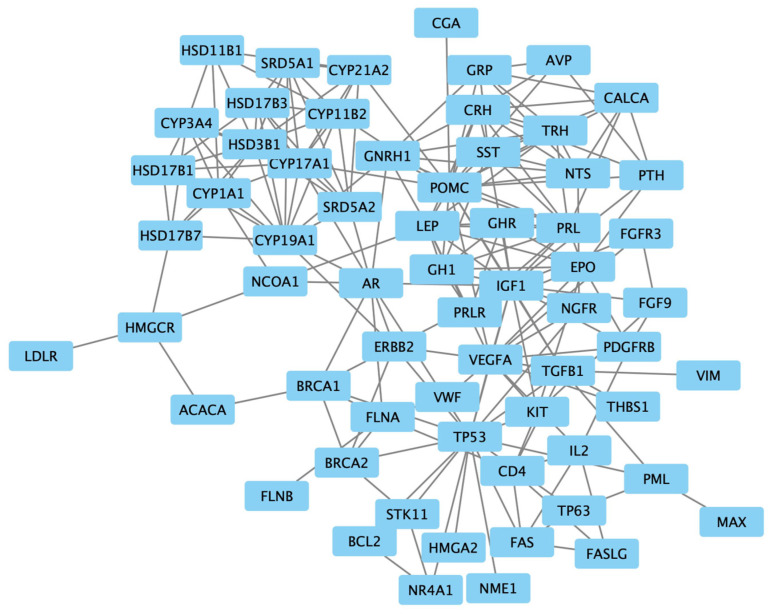
The protein–protein interaction network of 54 targeted genes, visualized using Cytoscape.

The examination of protein interactions followed the established approach of constructing a STRING protein–protein network (Figure 2). Within this network illustration, seven specific genes, namely *NODA*, *LALBA*, *ARHG*, *XCL1*, *ASS1*, *CAT* and *FPR1*, were distinctly set apart from the surrounding nodes, signifying their lack of interaction with other genes. The resultant PPI network, comprising a total of 54 genes, was then imported into Cytoscape for clear visualization (Figure 3).

Upon utilizing CentiScaPe, the average degrees and betweenness values within the protein–protein network were computed as 7.45 and 135.36, respectively. Recognizing the pronounced variability in betweenness values among nodes, a filtration process was enacted to exclude nodes with a betweenness value of 0, as they were deemed marginal. Subsequently, the remaining nodes, each possessing a minimum of two incident edges, were identified as the pivotal nodes in the network. As a result, a selection of 10 genes emerged from these key nodes, constituting the final list of genes. This exclusive list included ’insulin-like growth factor 1 (*IGF1*)’, ’transforming growth factor beta 1 (*TGFB1*)’, ’androgen receptor (*AR*)’, ’Cytochrome P450 Family 19 Subfamily A Member 1 (*CYP19A1*)’, ’Cytochrome P450 Family 17 Subfamily A Member 1 (*CYP17A1*)’, ’Proopiomelanocortin (*POMC*)’, ’Vascular Endothelial Growth Factor A (*VEGFA*)’, ’leptin (*LEP*)’ and ’gonadotropin-releasing hormone 1 (*GNRH1*)’.

### 3.3. Results of Drug–Gene Interactions

To investigate drug–gene interactions, the focus was directed towards the subset of 10 genes recognized as potential targets. A rigorous screening process was employed to exclude drugs originating from chemical synthesis that could be assessed using DeepPurpose. This process led to the identification of 21 drugs, each characterized by SMILES structures, forming the initial pool of candidate drugs. The subsequent step involved the application of DeepPurpose, utilizing 15 distinct prediction models, in order to establish the ultimate drug list associated with gynecomastia (Table 3).

Considering the diverse calculation approaches utilized for affinity scores across various datasets, specific affinity score thresholds were established. For models grounded in the DAVIS and BindingDB datasets, the affinity score threshold was defined as 7.0, while models utilizing the KIBA dataset employed a threshold of 12.1. Employing these thresholds, drugs were chosen if their binding affinity scores exceeded the designated threshold. Consequently, a refined drug list, comprising 12 compounds, was derived (Table 4). This comprehensive list of drugs includes conteltinib (targeting *IGF1R*), yifenidone (targeting *TGFB1*), vosilasarm (targeting *AR*), testosterone (targeting *AR*), cortexolone (targeting *AR*), CLAR-121 (targeting *AR*), dimethylcurcumin (targeting *AR*), FT-7051 (targeting *AR*), bremelanotide (targeting *POMC*), catequentinib (targeting *VEGFA*), vorolanib (targeting *VEGFA*), fenretinide (targeting *VEGFA*) and kevetrin (targeting *TP53*). 

## 4. Discussion

Gynecomastia is a relatively common disorder that has troubled men for a long time, posing potential health threats for them. However, due to the diversity of possible etiologies, the pathogenesis of gynecomastia remains unclear, and there is still no specific drug for its treatment.

This study identified 10 genes strongly associated with gynecomastia through data mining and 12 drugs targeting these genes using the DeepPurpose deep learning model. The potential drugs were categorized into *IGF1R* inhibitors, *ESR* agonists, *IGF-1* receptor tyrosine kinase inhibitors (*TKIs*) and *MMP1* inhibitors.

IGF-1 is a member of a family of proteins involved in mediating growth and development. Although estrogens and progestogens are vital to mammary growth, they are ineffective in the absence of IGF-1, and IGF-1 acts locally in the mammary gland to promote breast development [4]. A cohort study showed that increased IGF-1 levels are associated with gynecomastia in pubertal boys [18]. The GH/IGF-1 axis is also thought to interact with thyroid hormones, contributing to the development of pubertal gynecomastia [19]. Conteltinib, also called CT-707, is a novel multi-kinase inhibitor candidate that has been approved by the China Food and Drug Administration (CFDA) for phase I clinical trials. CT-707 serves mainly as an antitumor drug through inhibiting FAK or YAP [7,20]. However, recent research discovered CT-707 as a novel clinical approach for HCC through inhibiting IGF1R [21]. The study highlighted CT-707 as a small molecule inhibitor of IGF1R for the first time and also suggested that CT-707 may be a potential treatment for gynecomastia.

TGF-β regulates cell proliferation, differentiation and growth and can modulate expression and activation of other growth factors including IFN-γ and TNF-α. TGF-β is not only a regulator of normal mammary gland development by promoting branching morphogenesis but is also responsible for the progression of breast cancer [22]. An animal study demonstrated that an increase in TGF-β levels in transgenic male mice resulted in the induction of gynecomastia [23]. However, a cohort study showed no statistical difference in the expression of TGF-β receptors in patients with gynecomastia [22]. Yifenidone, a TGF-β antagonist, was developed mainly as treatment for fibrotic disease, but the results of DeepPurpose suggested that it may also have therapeutic effects on gynecomastia.

Gynecomastia is a prevalent condition frequently linked to an imbalance of estrogen and androgen levels, commonly arising from different endocrine disorders [24]. The disproportionate ratio of estrogen to androgen levels in tissues is considered a key factor in the development of gynecomastia [25]. As is well established, antiandrogens can block the effects of androgens on breast glandular tissue and disrupt the negative feedback mechanism of the hypothalamic–pituitary–gonadal axis, leading to increased androgen levels and promoting the development of gynecomastia [24]. Selective Androgen Receptor Modulators (SARMs) target the androgen receptor and represent potential alternatives for androgen supplementation. Therefore, androgen receptor modulators show potential therapeutic effects on gynecomastia. These drugs include vosilasarm, testosterone, cortexolone, CLAR-121, dimethylcurcumin and FT-7051. All of these drugs can be administered by injection, regulating the effect of androgens on local tissues, suggesting their potential for treating gynecomastia. However, hormone regulation is a relatively complex course. Further experimental research is required to evaluate their therapeutic efficacy. 

Peptides derived from proopiomelanocortin (POMC) are well-known neuropeptides and peptide hormones that exert various functions through enzymatic processes (PC1, PC2 and PC3) in a tissue-specific manner [26]. Among these, PC2 activity is particularly significant in the cutaneous processing of POMC [27]. Heterozygous variations in the POMC gene are relatively common and may contribute to obesity, which is strongly linked to gynecomastia [4]. The mechanism behind this connection is believed to involve increased aromatase activity in adipose tissue, indicating a potential role for POMC [28]. Bremelanotide, a melanocortin receptor agonist administered parenterally for the treatment of female hypoactive sexual desire disorder, may also be a viable option for treating gynecomastia.

VEGF is a positive regulator of angiogenesis, and its expression is upregulated in many types of cancers, including breast cancers. Experimental studies have shown that VEGF cytoplasmic reactivity is present in male breast cancer samples [29]. Tumor protein p53 is a critical regulator of multiple cellular pathways. Kevetrin has been developed for inhibiting p53-dependent activity in solid tumors [30]. However, there is currently no clear evidence linking VEGF or TP53 with gynecomastia [31]. Further research is needed to verify the therapeutic value of VEGF and TP53 receptor inhibitors for gynecomastia.

## 5. Conclusions

This study represents a novel application of computational tools, including text mining, pathway analysis and protein–protein interaction networks, combined with the DeepPurpose deep learning model, to identify potential therapeutic targets and candidate drugs for gynecomastia. Ten key genes associated with gynecomastia were identified, including *IGF1*, *TGFB1* and *AR*, alongside 12 candidate drugs with promising binding affinities, such as yifenidone, conteltinib and vosilasarm. Our findings highlight the potential therapeutic relevance of targeting pathways involved in growth factor signaling (e.g., IGF1R and TGFB1), androgen receptor modulation and melanocortin signaling for managing gynecomastia. While this study provides a foundational framework for drug discovery, further experimental and clinical studies are essential to validate these computational predictions. Future research will focus on optimizing prediction models and advancing preclinical investigations to bridge the gap between computational insights and clinical application.

## Figures and Tables

**Table 1:** Summary of gene set enrichment analysis for biological process.

Description	Number of Genes	Corrected Hypergeometric *p* Value	Genes
signal transduction	40	3.67 × 10^−23^	*FPR1*, *LALBA*, *CD4*, *TRH*, *NGFR*, *GPR182*, *PDGFRB*, *PRL*, *HMGA2*, etc.
positive regulation of cell population proliferation	26	1.98 × 10^−21^	*MECP2*, *TGFB1*, *IL2*, *PDGFRB*, *PRL*, *FGF9*, *CGA*, *AR*, *EPO*, *ERBB2*, etc.
cell–cell signaling	20	4.01 × 10^−21^	*LALBA*, *TRH*, *IL2*, *FGF9*, *PTH*, *XCL1*, *GNRH1*, *AR*, *FGFR3*, *CGB8*, etc.
steroid biosynthetic process	14	1.90 × 10^−20^	*CYP1A1*, *CYP11B2*, *HSD17B7*, *CYP19A1*, *CYP17A1*, *SRD5A1*, etc.
steroid metabolic process	15	4.82 × 10^−18^	*APOA1*, *CYP1A1*, *CYP11B2*, *CYP17A1*, *SRD5A1*, *CYP21A2*, *CYP3A4*, etc.
negative regulation of apoptotic process	23	4.90 × 10^−18^	*IL2*, *NGFR*, *PDGFRB*, *HMGA2*, *GNRH1*, *TP53*, *EPO*, *MGMT*, *BCL2*, etc.
response to estradiol	14	4.24 × 10^−17^	*TGFB1*, *PDGFRB*, *CYP19A1*, *GH1*, *SRD5A1*, *ASS1*, *NCOA1*, *PCNA*, etc.
response to drug	18	1.69 × 10^−16^	*APOA1*, *CYP1A1*, *ABCC6*, *PTH*, *SRD5A1*, *ASS1*, *TP53*, *MGMT*, etc.
positive regulation of gene expression	21	4.15 × 10^−16^	*TGFB1*, *PLAG1*, *HMGA2*, *FGF9*, *CD34*, *BRCA1*, *AR*, *SRY*, *TP53*, *VIM*, etc.
aging	15	5.35 × 10^−16^	*CYP1A1*, *MME*, *PDGFRB*, *IGFBP1*, *GNRH1*, *ASS1*, *EPO*, *GJB2*, etc.

**Table 2:** Summary of Kyoto Encyclopedia of Genes and Genomes (KEGG) process gene set enrichment analysis.

Description	Numbers of Genes	Genes
PI3K-Akt signaling pathway	22	*IL2*, *NGFR*, *PDFRB*, *PRL*, *FGF9*, *GH1*, *STK11*, *BRCA1*, *TP53*, *EPO*, *ERBB2*, *FGFR3*, *KIT*, *BCL2*, *VWF*, *FASLG*, etc.
Steroid hormone biosynthesis	13	*CYP1A1*, *CYP11B2*, *HSD17B7*, *CYP19A1*, *CYP17A1*, *SRD5A1*, *CYP21A2*, *HSD17B1*, *CYP3A4*, *HSD3B1*, etc.
Neuroactive ligand–receptor interaction	17	*FPR1*, *TRH*, *PRL*, *GH1*, *PTH*, *CGA*, *GNRH1*, *NTS*, *GRP*, *AVP*, *LEP*, *SST*, *POMC*, *CRH*, *GHR*, *PRLR*, *CALCA*
Metabolic pathways, steroid hormone biosynthesis	11	*CYP1A1*, *CYP11B2*, *HSD17B7*, *CYP19A1*, *CYP17A1*, *CYP21A2*, *HSD17B1*, *CYP3A4*, *HSD3B1*, *HSD11B1*, *HSD17B3*
Pathways in cancer	20	*TGFB1*, *MAX*, *IL2*, *PDFRB*, *FGF9*, *NCOA1*, *AR*, *TP53*, *EPO*, *ERBB2*, *ARHGEF1*, *FGFR3*, *KIT*, *BCL2*, *BRCA2*, etc.
MAPK signaling pathway	16	*TGFB1*, *MAX*, *NGFR*, *PDFRB*, *FGF9*, *TP53*, *ERBB2*, *FGFR3*, *KIT*, *FASLG*, *FLNB*, *FLNA*, *FAS,* etc.
Cytokine–cytokine receptor interaction	14	*TGFB1*, *CD4*, *IL2*, *NGFR*, *PRL*, *GH1*, *XCL1*, *EPO*, *FASLG*, *LEP*, *FAS*, *NODAL*, *GHR*, *PRLR*
Pathways in cancer, MAPK signaling pathway	12	*TGFB1*, *MAX*, *PDFRB*, *FGF9*, *TP53*, *ERBB2*, *FGFR3*, *KIT*, *FASLG*, *FAS*, *IGF1*, *VEGFA*
PI3K-Akt signaling pathway, MAPK signaling pathway	11	*NGFR*, *PDFRB*, *FGF9*, *TP53*, *ERBB2*, *FGFR3*, *KIT*, *FASLG*, *IGF1*, *VEGFA*, *NR4A1*
Pathways in cancer, PI3K-Akt signaling pathway	12	*IL2*, *PDFRB*, *FGF9*, *TP53*, *EPO*, *ERBB2*, *FGFR3*, *KIT*, *BCL2*, *FASLG*, *IGF1*, *VEGFA*

**Table 3:** Identification of potential candidate drugs for gynecomastia treatment using DeepPurpose.

Drug Name	Target Gene	Specific Target
conteltinib	*IGF1*	insulin like growth factor 1
pirfenidone, GNI	*TGFB1*	transforming growth factor, beta 1
pirfenidone, gel, CellPharma	*TGFB1*	transforming growth factor, beta 1
pirfenidone, extended release, CellPharma	*TGFB1*	transforming growth factor, beta 1
yifenidone, HEC Pharm	*TGFB1*	transforming growth factor, beta 1
timbetasin	*TGFB1*	transforming growth factor, beta 1
tranilast	*TGFB1*	transforming growth factor, beta 1
vosilasarm	*AR*	Androgen receptor
testosterone	*AR*	Androgen receptor
cortexolone	*AR*	Androgen receptor
CLAR-121	*AR*	Androgen receptor
dimethylcurcumin	*AR*	Androgen receptor
FT-7051	*AR*	Androgen receptor
letrozole	*CYP19A1*	Cytochrome P450 Family 19 Subfamily A Member 1
CLAR-121	*CYP19A1*	Proopiomelanocortin
bremelanotide	*POMC*	Proopiomelanocortin
catequentinib	*VEGFA*	Vascular Endothelial Growth Factor A
vorolanib	*VEGFA*	Vascular Endothelial Growth Factor A
FN-1501	*VEGFA*	Vascular Endothelial Growth Factor A
fenretinide	*VEGFA*	Vascular Endothelial Growth Factor A
CBL-0137	*TP53*	TP53
kevetrin	*TP53*	TP53

**Table 4:** Final list of candidate drugs targeting genes strongly associated with gynecomastia.

Gene	DeepDTA_DAVIS	Morgan_CNN_DAVIS	MPNN_CNN_DAVIS	Daylight_AAC_DAVIS	Morgan_AAC_DAVIS	CNN_CNN_BindingDB	Morgan_CNN_BindingDB	MPNN_CNN_BindingDB	Transformer_CNN_BindingDB	Daylight_AAC_BindingDB	Morgan_AAC_BindingDB	Morgan_CNN_KIBA	MPNN_CNN_KIBA	Daylight_AAC_KIBA	Morgan_AAC_KIBA
*IGF1*	5.2	5.6	6.6	6.1	5.3	7.2	6.7	5.4	5.9	5.4	5.1	11.5	11.4	11.5	11.7
*IGF1*	5.1	5.5	6.4	6	5.3	7.3	6.7	5.4	5.8	5.4	5.1	11.5	11.5	11.5	11.7
*TGFB1*	5	5	3.3	5.1	5.1	4.8	4	5.3	5.9	4.6	3.8	11.4	10.9	10.7	11.5
*TGFB1*	5	5	3.3	5.1	5.1	4.8	4	5.3	5.9	4.6	3.8	11.4	10.9	10.7	11.5
*TGFB1*	5	5	3.3	5.1	5.1	4.8	4	5.3	5.9	4.6	3.8	11.4	10.9	10.7	11.5
*TGFB1*	5.4	5	7.2	5.4	5.1	6.6	6.1	5.6	6.2	7	5.7	11.4	12.3	11.5	11.5
*TGFB1*	5.1	5.4	4.8	5.1	5.1	6.1	6.2	5.5	6.3	4.9	5	10.7	10.5	11.4	11.1
*TGFB1*	4.9	5	5.7	5	5.1	5.4	5	5.4	6.8	4.8	3.8	11.9	11.7	11.7	11.7
*AR*	5.3	5.1	8.6	5.2	5.2	7	7	5.5	6.7	6.7	5.2	11.3	10.9	11.2	11.6
*AR*	5.3	5.5	9.3	5.5	5.1	8	9	6.2	7.9	6.5	6.7	11	10.7	10.4	10.8
*AR*	5.5	5.3	9	5.4	5.1	7.7	7.8	7.4	7.9	5.3	5.4	11.1	10.9	10.3	10.8
*AR*	5.3	5.5	9.3	5.5	5.1	8	9	6.2	8	6.5	6.7	11	10.7	10.4	10.8
*AR*	5	5.1	8.3	5	5.1	7	4.8	5.8	7.3	4.4	4.3	11.8	11.9	11.4	11.5
*AR*	5.6	5.1	8.9	5.6	5.1	8.3	6.5	5.5	8	5.5	5.1	11.4	11.8	11.4	11.6
*CYP19A1*	4.9	5	5.4	5	5.1	6	4.5	5.2	6.1	5	3.3	11.3	11.3	11.2	11.5
*CYP19A1*	5.2	5.7	6.4	5.5	5.1	6.8	8.8	6.2	7.7	6.5	6.7	11	10.7	10.4	10.8
*CYP19A1*	5.3	5.4	6.5	5.5	5.1	6.7	9.1	6.2	7	6.5	6.7	11	10.7	10.4	10.8
*POMC*	5.5	5.1	4.7	5	5.1	5.7	9	5.2	5.7	5.2	5.8	11	11.4	10.4	11.7
*VEGFA*	4.7	5	6.8	5.1	5	7.9	5.1	5.4	5.4	5	5	11.3	11.3	10.6	11.5
*VEGFA*	5.5	5.8	4.9	5	5.1	4.3	6.7	5.8	7.5	5	5	11.6	11.7	11.8	11.6
*VEGFA*	4.9	5.1	5.1	5.6	5	4.4	4.5	5.2	6.3	5.5	4.9	11.3	11.5	11.5	11.3
*VEGFA*	4.9	5.1	6	5.8	5.1	6.4	7.6	5.4	7.5	5.1	5.3	12.9	11.2	11.5	11.7
*TP53*	4.9	5.1	5.3	5.2	5.1	6.1	5.6	5.2	6.6	5	4.6	11.4	11.4	11.5	11.1
*TP53*	5	5	3.6	5	5.1	3.8	4.3	4.9	4.6	4.4	4.2	11.9	13.8	10.2	12.2

## Data Availability

The original contributions presented in this study are included in the article. Further inquiries can be directed to the corresponding authors.
